# Antioxidants in neuropsychiatric disorder prevention: neuroprotection, synaptic regulation, microglia modulation, and neurotrophic effects

**DOI:** 10.3389/fnins.2024.1505153

**Published:** 2024-12-05

**Authors:** Fangfei Liu, Qianqian Bai, Wenchao Tang, Shumin Zhang, Yan Guo, Shunji Pan, Xiaoyu Ma, Yanhui Yang, Hua Fan

**Affiliations:** ^1^The First Affiliated Hospital, College of Clinical Medicine of Henan University of Science and Technology, Luoyang, China; ^2^Department of Trauma Center, The First Affiliated Hospital of Henan University of Science and Technology, Luoyang, China; ^3^Office of Research and Innovation, The First Affiliated Hospital of Henan University of Science and Technology, Luoyang, China

**Keywords:** oxidative stress, neuropsychiatric disorders, antioxidants, neuroprotection, synaptic regulation, microglia modulation, neurotrophic effects

## Abstract

Oxidative stress, caused by an imbalance between the generation of reactive oxygen species (ROS) and the body’s intrinsic antioxidant defenses, plays a critical role in neurodegenerative diseases such as Alzheimer’s, Parkinson’s, and Huntington’s. Beyond these conditions, recent evidence indicates that dysregulated redox balance is implicated in neuropsychiatric disorders, including schizophrenia, major depressive disorder, and anxiety disorders. Preclinical and clinical studies have demonstrated the potential of antioxidants, such as N-acetylcysteine, sulforaphane, alpha-lipoic acid, L-carnitine, ascorbic acid, selenocompounds, flavones and zinc, in alleviating neuropsychiatric symptoms by mitigating excitotoxicity, enhancing synaptic plasticity, reducing microglial overactivation and promoting synaptogenesis. This review explores the role of oxidative stress in the pathogenesis of neuropsychiatric disorders. It provides an overview of the current evidence on antioxidant therapy’s pharmacological effects, as demonstrated in animal models and clinical studies. It also discusses the underlying mechanisms and future directions for developing antioxidant-based adjuvant therapies. Given the limitations and side effects of existing treatments for neuropsychiatric disorders, antioxidant therapy presents a promising, safer alternative. Further research is essential to deepen our understanding and investigate the clinical efficacy and mechanisms underlying these therapies.

## 1 Oxidative stress and brain disorders

The brain relies heavily on oxygen to generate the energy required for cognitive function. The release of neurotransmitter-loaded vesicles at synapses demands substantial energy, with approximately 1.64 × 10^5 ATP molecules needed per vesicle released (Alle et al., 2009; Magistretti and Allaman, 2015). Consequently, neuronal mitochondria must consume oxygen (O_2_) at a disproportionately high rate to meet the brain’s energy needs (Alle et al., 2009). The brain depends on O_2_ for aerobic respiration to sustain its high metabolic activity; however, this process produces reactive oxygen species (ROS) as byproducts, including superoxide anions (O_2_⁻), hydrogen peroxide (H_2_O_2_), and hydroxyl radicals (·OH), alongside the complete reduction of oxygen to water (Lennicke and Cocheme, 2021). Under normal physiological conditions, ROS participates in cellular signaling, regulating cell growth and maintaining homeostasis. For instance, low concentrations of O_2_⁻ and H_2_O_2_ can stimulate the proliferation of adult hippocampal progenitor cells (Dickinson et al., 2011). However, when oxidative stress (OS) overwhelms the body’s antioxidant defenses, excessive ROS can damage neurons, contributing to the development of neurodegenerative (Shadfar et al., 2023) and neuropsychiatric diseases (Rossetti et al., 2020).

While the body’s antioxidant enzymes typically neutralize peroxidation products, ROS have evolved to fulfill critical physiological roles, especially within the central nervous system. Consequently, the brain’s antioxidant system must make certain compromises (Murphy et al., 2011). For instance, neurons contain significantly lower levels of catalase (CAT) (approximately 50 times less than liver cells) (Ren et al., 2017) and approximately half the amount of cytoplasmic glutathione (GSH) compared to liver cells (Paul et al., 2018; Cobley et al., 2018). This relatively weak endogenous antioxidant defense makes the brain particularly vulnerable to OS (Cobley et al., 2018).

Moreover, the brain’s neuronal membranes are rich in unsaturated fatty acids, making them susceptible to oxidative damage, which can produce reactive aldehydes (Maiorino et al., 2018). During immune responses, microglia release substances such as O_2_⁻ and ROS (Block et al., 2007). Furthermore, H_2_O_2_ is produced during the metabolism of neurotransmitters (Ren et al., 2017). Mitochondrial dysfunction further exacerbates OS by increasing ROS production, creating a vicious cycle in neuronal cells reliant on mitochondrial activity (Rizzuto et al., 2012; Slimen et al., 2014).

The brain’s vulnerability to OS stems from several factors, including its high metabolic demands, relatively weak antioxidant defenses, and abundant unsaturated fatty acids in neuronal membranes. These characteristics suggest that OS plays a pivotal role in the pathogenesis of neurological and psychiatric disorders.

## 2 The role of oxidative stress in neuropsychiatric disorders

The brain is particularly susceptible to OS, and its role in the pathogenesis of neuropsychiatric disorders has gained increasing attention in recent years (Rossetti et al., 2020). Therefore, this section reviews the evidence linking OS to conditions such as schizophrenia (SZ), anxiety disorders, major depressive disorder (MDD) and bipolar disorder (BD).

### 2.1 Oxidative stress in schizophrenia

SZ is a severe mental disorder affecting approximately 0.3 to 0.66% of the population, significantly impairing quality of life and imposing a substantial socio-economic burden (Maas et al., 2017).

While traditional models of SZ pathogenesis emphasize neurotransmitter dysfunction, particularly involving dopamine, emerging research points to OS as an additional underlying mechanism (Miljevic et al., 2018). This hypothesis is supported by numerous studies and meta-analyses (Goh et al., 2021; Goh et al., 2022). For instance, research by Li et al. (2024) and Chien et al. (2020) has shown significantly elevated levels of malondialdehyde (MDA), a marker of lipid peroxidation (LP), in the blood samples of patients with SZ. Similarly, a study by Jia et al. (2023) indicated that OS contributes to hippocampal damage in patients with first-episode SZ, leading to cognitive impairment. Raffa et al. (2011) identified reduced activity of antioxidant defense systems, such as GSH and CAT, in individuals with SZ. Further evidence from Al-Amin et al. (2016) suggests that the antioxidant astaxanthin can ameliorate behavioral deficits in SZ mice. Concurrently, MacDowell et al. (2016) suggested that the antipsychotic drug paliperidone may mitigate OS by upregulating nuclear factor erythroid 2-related factor 2 (Nrf2) in the Phosphoinositide 3-kinase/Protein kinase B (PI3K/AKT) pathway. Kulak et al. (2013) observed heightened OS in the anterior cingulate cortex during early development in GSH synthesis-deficient (gclm −/−) mice accompanied by microglial activation and redox-sensitive matrix metalloproteinase 9 (MMP9) upregulation. Inhibiting MMP9 activation can normalize parvalbumin-expressing interneurons (PVI)/ perineuronal nets (PNN) maturation and alleviate SZ-related psychopathology (Dwir et al., 2020).

Furthermore, extensive research suggests that OS may impact cognitive function through various pathways, such as directly damaging parvalbumin-expressing interneurons (PVIs) (Schiavone et al., 2009), hindering oligodendrocyte precursor cell (OPC) proliferation and myelin formation in the prefrontal cortex (PFC) (Maas et al., 2021), disrupting the blood–brain barrier (BBB) (Geng et al., 2023), and inducing mitochondrial dysfunction (Fizikova et al., 2023). Therefore, targeting OS may be crucial for SZ prevention and treatment.

### 2.2 Oxidative stress in major depressive disorder

According to the World Health Organization (WHO), MDD was the fourth leading cause of disability worldwide and was predicted to rise to second by 2020. Nearly half of those affected may not receive timely diagnosis and treatment, underscoring the urgent public health challenge of managing depression (Lolak et al., 2014).

Traditional models attribute depression to disruptions in monoamine and glutamate neurotransmission. However, emerging evidence suggests that OS and pro-inflammatory signaling may also contribute to MDD (Bader et al., 2024; Tuon et al., 2021). Jiménez and Chung et al. found significantly elevated levels of MDA in the plasma of patients with MDD (Jimenez-Fernandez et al., 2022; Chung et al., 2013). Similarly, Maes et al. (2019) reported increased levels of superoxide dismutase 1 (SOD1), nitric oxide (NO), ROS, and lipid peroxides in patients with depressive symptoms. Conversely, Kotan et al. (2011) identified decreased activity of antioxidant enzymes, such as SOD and CAT, in the serum of patients with MDD. Szebeni et al. (2014) reported significantly reduced mRNA levels of SOD, CAT, and glutathione peroxidase (GPX) in oligodendrocytes from the white matter of patients with MDD in post-mortem analysis. Moreover, Moreno et al. (2013) found elevated platelet NO and platelet mitochondrial membrane potential (PMMP) in patients with MDD, suggesting that mitochondrial bioenergetic alterations may contribute to the onset and progression of depression via OS. This evidence is further supported in animal models of depression (Tuon et al., 2021). Moreover, knockout (KO) mice lacking the antioxidant transcription factor Nrf2 displayed depression-like behaviors in various tests (Dang et al., 2022; Zeng et al., 2023).

OS may disrupt neurotransmitter metabolism, such as that of serotonin (Ding et al., 2020), impair neurogenesis and synaptic plasticity (Hou et al., 2017), and induce DNA and RNA hypermethylation (Wu et al., 2021; Han et al., 2022), all of which may contribute to depression. These findings underscore the therapeutic potential of antioxidants in treating depression.

### 2.3 Oxidative stress in anxiety disorders

Anxiety, an essential evolutionary mechanism for alertness and self-protection, can become maladaptive when excessive, leading to anxiety disorders. The lifetime prevalence of pathological anxiety exceeds 20% (Filiou and Sandi, 2019; Koskinen and Hovatta, 2023).

Anxiety disorders, including generalized anxiety disorder (GAD) and phobias, are not fully understood. However, emerging research hints at a potential role for impaired antioxidant defense and oxidative damage in their development (Kaya et al., 2013; Oktay et al., 2024). Oktay et al. (2024) clinical study revealed significantly increased levels of LP markers, such as MDA and F2-isoprostanes, in patients with severe anxiety. Bellisario et al. (2014) demonstrated that deleting the p66Shc gene, a key regulator of mitochondrial ROS production, reduced anxiety behaviors by reducing OS. Furthermore, Bersuker et al. (2019) discovered that *Lactobacillus plantarum* guanidinoacetate (LbGp), an OS regulator, alleviated anxiety-like behavior by enhancing glutathione peroxidase 4 (GPX4) activity and preventing ferroptosis. Conversely, the deletion of the GPX4 gene in dopaminergic neurons increased anxiety behaviors (Dang et al., 2022). Moreover, overexpression of genes such as glutathione reductase 1 (GSR1) and glyoxalase enzyme 1 (GLO1) has been strongly correlated with anxiety phenotypes (Hovatta et al., 2005), with GLO1 inhibitors showing potential in alleviating anxiety (Distler et al., 2012). Moreover, OS may exacerbate anxiety by depleting reduced GSH (Nisar et al., 2023) and promoting N-methyl-D-aspartate (NMDA) receptor-mediated synaptic inhibition in the basolateral amygdala (BLA) (Wu et al., 2022).

Despite inconsistent findings across studies, a general pattern of oxidative imbalance has been observed in patients with anxiety, suggesting that targeting OS may offer a promising therapeutic avenue for anxiety disorders.

### 2.4 Oxidative stress in bipolar disorder

Bipolar disorder (BD) is a chronic mental illness characterized by an alternation between mania or hypomania and depression. It is often associated with impaired functionality (Munkholm et al., 2024).

Several lines of evidence point to the presence of low-grade inflammation and oxidative stress in patients with bipolar disorder (Rosenblat and McIntyre, 2016), while findings to some extent are inconsistent and have been limited by methodological issues (Garcia-Gutierrez et al., 2020; Kirkpatrick et al., 2021; Munkholm et al., 2024). Increased lipid peroxidation has been observed in the prefrontal cortex and anterior cingulate cortex of patients with BD (Wang et al., 2009). Moreover, One study conducted with 94 BD patients and 41 healthy controls reported higher OS index levels in the BD patients compared with the controls (Yumru et al., 2009). It also found decreased antioxidant and OS markers; however, many other studies have reported the opposite finding. For example, some studies corroborated this finding of increased serum TBARS levels in BD patients during mania, depression, and euthymia (Andreazza et al., 2007). Moreover, Sowa-Kucma et al. (2018) found a significant positive association between higher TBARS level and severity of BD, including the risk of suicidality. Additionally, studies have found that serum copper concentrations may be higher in certain subgroups, such as patients in the early stages of the disease. Furthermore, serum copper concentrations may be associated with certain pathophysiological processes of bipolar disorder, such as oxidative stress. Although this study suggests that there are differences in serum copper concentrations among bipolar disorder patients at different stages of the disease, these differences did not reach statistical significance (Siwek et al., 2017).

BD is becoming increasingly understood as a condition of aberrant neuroplasticity. Multiple factors, such as OS, imbalance of neurotransmitters, and genetics, are associated with the pathophysiology of BD.

## 3 The role of antioxidants in treating neuropsychiatric disorders

**Figure 1:**
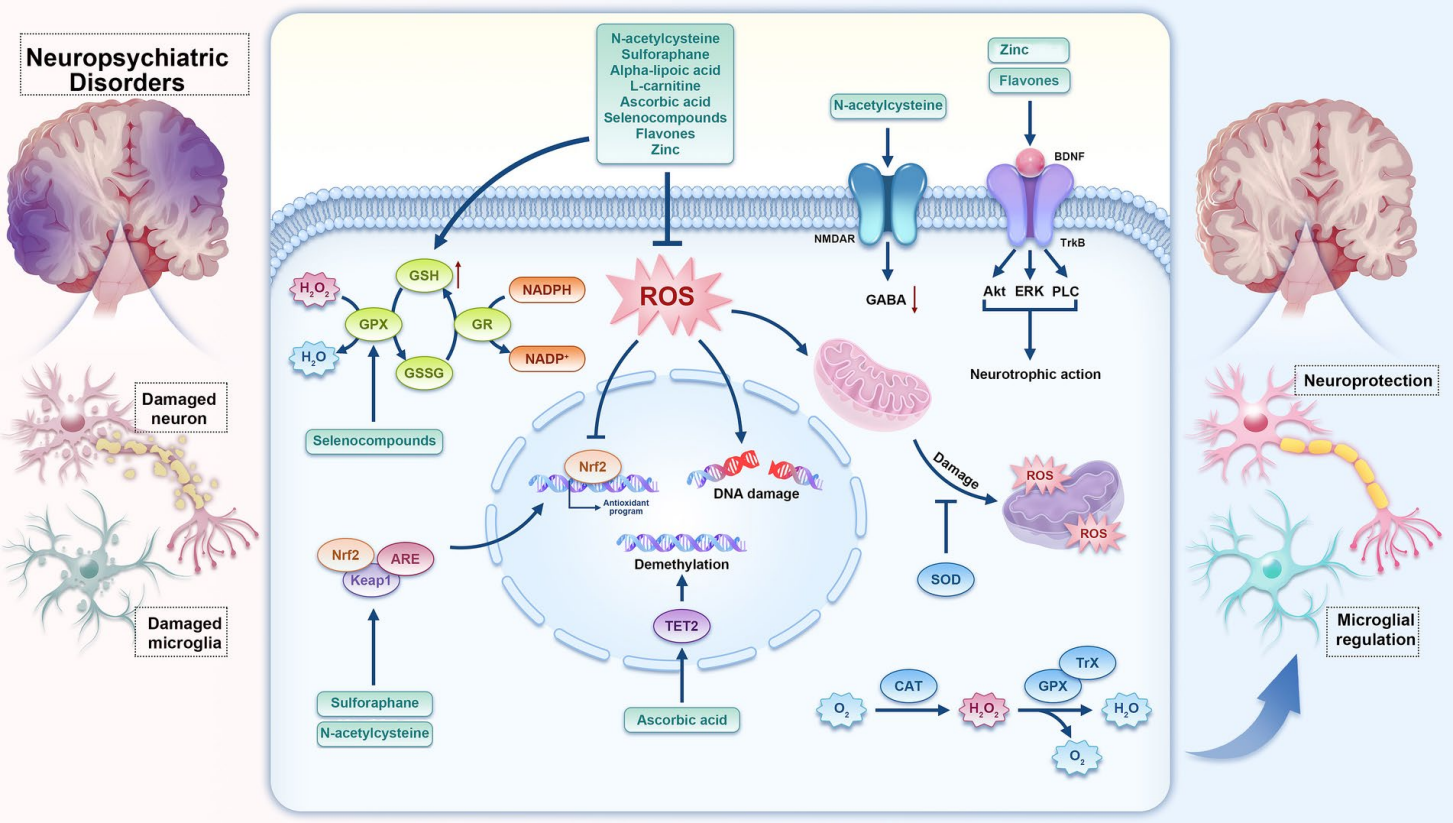
The hypothesis map illustrates the interplay between natural antioxidant compounds, the body’s innate antioxidant defenses, reactive oxygen species (ROS), microglial inflammatory responses, and the management of neuropsychiatric disorders. Antioxidants such as N-acetylcysteine, sulforaphane, alpha-lipoic acid, L-carnitine, ascorbic acid, selenocompounds, flavones, and zinc, etc., not only function by scavenging ROS, enhancing the activity of antioxidant enzymes, such as superoxide dismutase (SOD), glutathione peroxidase (GPx), catalase (CAT), and thioredoxin (Trx), and promoting the expression of antioxidant genes such as nuclear factor erythroid 2-related factor 2 (Nrf2), but they also exert effects on specific molecular targets. For instance, N-acetylcysteine (NAC) and zinc can directly interact with N-methyl-D-aspartate (NMDA) receptors, ascorbic acid can directly affect the activity of Tet Methylcytosine Dioxygenase 2 (TET2) enzymes, and Flavonoid compounds have the ability to directly interact with tyrosine kinase receptor B (TrkB) receptors and subsequently activate downstream signaling pathways such as Phospholipase C (PLC), Extracellular Signal-Regulated Kinase (ERK), and Protein kinase B (AKT).

The antioxidant system of cells is mainly composed of two parts: the enzymatic antioxidant system and the non-enzymatic antioxidant system. These two systems are not isolated but form an integral whole. The enzymatic antioxidant system includes a series of active enzymes with antioxidant properties, such as superoxide dismutase (SOD, including Cu-Zn SOD and Mn-SOD), catalase (CAT), glutathione peroxidase (GPx), thioredoxin (Trx), and others (Wen et al., 2022; Chen et al., 2023). These enzymes can catalyze antioxidant reactions, converting free radicals into harmless substances, thereby maintaining redox balance within organisms. The non-enzymatic antioxidant system, on the other hand, is primarily composed of small molecular antioxidant substances. Numerous preclinical and clinical studies highlight the potential of antioxidants (Rossetti et al., 2020) such as N-acetylcysteine (NAC), sulforaphane (SFN), alpha-lipoic acid (ALA), L-carnitine (L-Car), ascorbic acid, selenocompounds, and flavones. Beyond their direct radical-scavenging properties, these compounds have demonstrated an ability to modulate endogenous antioxidant systems. (Figure 1).

### 3.1 N-acetylcysteine

N-acetylcysteine (NAC), an essential precursor for GSH synthesis, is a critical brain antioxidant (Raghu et al., 2021). Its antioxidant mechanisms primarily encompass: serving as a reductant to reduce oxidized molecules by donating electrons; activating the glutathione (GSH) cycle to restore glutathione to its reduced form; directly scavenging free radicals, including superoxide anions, hydroxyl radicals, and hydrogen peroxide; and curbing inflammation by suppressing oxidative stress and inflammatory cytokine production (Raghu et al., 2021). Several studies have suggested that NAC can ameliorate clinical symptoms in patients with SZ, AN, and MDD (Sceneay et al., 2013; Hoepner et al., 2021). For instance, NAC modulates GSH and glutamate levels, potentially reducing the negative symptoms and cognitive impairments associated with SZ (Yang et al., 2022). However, while evidence supports NAC’s therapeutic effect in stable patients with SZ, its efficacy in patients with refractory SZ on clozapine remains inconclusive (Fornaro et al., 2024). Furthermore, animal experiments indicate that NAC can mitigate elevated glutamate levels in the cerebral cortex, reduce ROS levels in interneurons (Neill et al., 2022; Buhner et al., 2022), and upregulate brain-derived neurotrophic factor (BDNF) mRNA and protein, leading to improved behavioral and cognitive outcomes in SZ animal models (Phensy et al., 2017; Aslanlar et al., 2024). Similarly, NAC has been shown to alleviate moderate depressive symptoms (Liang et al., 2022) by curbing ROS production in microglia (Lehmann et al., 2019) and regulating the glutamatergic system in the PFC (Nery et al., 2022). However, a meta-analysis of randomized controlled trials found that NAC was not significantly better than placebo in treating severe depression or bipolar disorder (Andrade, 2021).

Overall, NAC exhibits multiple biological activities, demonstrating promise as a treatment for SZ, MDD, and AN; however, further research is warranted.

### 3.2 Sulforaphane

Sulforaphane (SFN) is a naturally occurring organic sulfur compound found in cruciferous vegetables such as broccoli, cauliflower, and mustard greens, characterized by its unique isothiocyanate group (Kamal et al., 2020). As an indirect antioxidant, SFN activates the Nrf2/Kelch-like ECH-associated protein 1 (Keap1)/Antioxidant response element (ARE) signaling pathway. When cells are stimulated by oxidative stress or other stressors, SFN binds to specific sites on Keap1, causing a conformational change in Keap1. This change frees Nrf2 from its binding with Keap1, allowing it to translocate to the nucleus. In the nucleus, Nrf2 binds to ARE, initiating the transcription of a series of antioxidant enzymes and proteins, thereby preserving cellular redox balance and homeostasis (Mangla et al., 2021). Additionally, by activating the Nrf2/Keap1/ARE signaling pathway, SFN upregulates the activity of multiple antioxidant enzymes, protecting cells from oxidative damage (Ma et al., 2023). Beyond its antioxidant effects, SFN exhibits potent anti-inflammatory properties (Kiser et al., 2021).

Some clinical studies indicate that SFN can prevent cognitive impairment in SZ through its anti-inflammatory (Zeng et al., 2024) and antioxidant effects (Shirai et al., 2015). However, other trials have not consistently replicated these findings (Dickerson et al., 2021). In animal models, SFN appears to be a promising adjunct therapy for SZ, mitigating side effects such as metabolic defects, biochemical imbalances, and liver histological abnormalities associated with olanzapine (OLA) (El-Shoura et al., 2024). Concurrently, SFN has been shown to improve anxiety and depression symptoms in mice by activating the Nrf2/ heme oxygenase-1 (HO-1) signaling pathway (Ferreira-Chamorro et al., 2018) and inhibiting the hypothalamic–pituitary–adrenal (HPA) axis and stress response (Wu et al., 2016). Furthermore, SFN may activate Nrf2 by initiating the transcription of trigger receptor expressed on myeloid cells-2 (TREM2) in the medial PFC (mPFC), increasing the expression of the M2 microglial marker arginase 1 (ARG1), which may alleviate depressive phenotypes through its anti-inflammatory and neuroprotective functions (He et al., 2022).

In summary, SFN has demonstrated potential as a treatment for SZ, MDD, and AN. However, further high-quality clinical and animal studies are necessary to confirm its therapeutic efficacy and mechanisms of action.

### 3.3 Alpha-lipoic acid

Alpha-lipoic acid (ALA) is a natural compound commonly found in the diet, serving as a crucial cofactor for mitochondrial respiratory enzymes and playing a vital role in maintaining cellular oxidative metabolism (Holmquist et al., 2007). ALA can directly scavenge ROS, promote the regeneration of vitamins C and E, and upregulate the activity of antioxidant enzymes like superoxide dismutase and catalase (El-Houseiny et al., 2023). Evidence suggests that ALA may alleviate symptoms associated with SZ and reduce OS (Emsley et al., 2014; Vasconcelos et al., 2015). Furthermore, ALA supplementation has been shown to improve the psychopathology of patients with treatment-resistant SZ (TRS) by decreasing OS (Sanders et al., 2017; Mishra et al., 2022). However, these promising findings were not confirmed in a subsequent double-blinded, placebo-controlled trial conducted by Emsley et al. (2014), warranting caution due to potential side effects, including a decrease in blood cell count associated with ALA treatment (De Lima et al., 2023). Iannuzzo et al. (2022) investigated ALA’s potential for treating depression, particularly in combination with other therapies, as it can effectively mitigate drug-related side effects such as the risk of diabetes and liver dysfunction. Moreover, ALA has been demonstrated to regulate the neuropathology of BDNF in mice model (Vasconcelos et al., 2015; Aliomrani et al., 2022). Furthermore, ALA alleviates methamphetamine-induced memory deficits and anxiety-like behavior in rats by enhancing the activity of antioxidant enzymes, including SOD and CAT (Kargar and Noshiri, 2024).

These findings underscore ALA’s potential to enhance cognitive function and emotional well-being while highlighting the necessity for further clinical validation in human populations.

### 3.4 L-carnitine

L-Carnitine (L-Car) is an essential nutrient in human tissues, including the brain. The antioxidant mechanism of L-Car primarily involves facilitating fatty acid entry into mitochondria for oxidative breakdown, reducing intracellular fatty acid accumulation, stabilizing mitochondrial membrane potential, scavenging free radicals, upregulating the expression of antioxidant enzyme genes, and enhancing antioxidant enzyme activity (Da Silva et al., 2023). These actions collectively protect cells from damage caused by oxidative stress. Specifically, acetyl-L-Car (ALCAR), as a critical form of L-Car, has been substantially linked to several mental health disorders (Cao et al., 2019). Previous studies indicate that low levels of ALCAR are closely associated with conditions such as depression and SZ (Cao et al., 2020). L-Car has been shown to improve psychiatric scores in a mouse model of SZ through anti-inflammatory and antioxidant pathways (Ebrahimi et al., 2023). Meanwhile, clozapine can disrupt lipid metabolism in the liver by affecting L-Car reabsorption, and concurrent L-Car supplementation is an effective strategy to mitigate these metabolic disturbances (Bruno et al., 2016; Wang et al., 2018). Moreover, metabolomic analyses of serum from patients with severe depression suggest that L-Car and ALCAR may serve as potential biomarkers for this condition (Nie et al., 2021). Supplementation with L-Car may serve as an effective adjuvant therapy for patients with refractory depression. The Canadian Emotion and Anxiety Treatment Network has established clinical guidelines recommending ALCAR monotherapy as a third-line treatment option for mild to moderate depression based on existing research evidence (Yatham et al., 2018). A recent meta-analysis showed that ALCAR supplementation as a standalone intervention significantly alleviated depressive symptoms compared to placebo or no intervention (Veronese et al., 2018). Animal studies suggest that ALCAR may exert antidepressant effects through the PI3K/AKT/BDNF signaling pathway (Wang et al., 2015).

Although ALCAR’s potential in treating mental illness has been preliminarily validated, further high-quality research is necessary to explore its specific mechanisms and optimize treatment dosages and regimens. Moreover, attention must be paid to the interactions between ALCAR and other medications and their potential adverse reactions.

### 3.5 Ascorbic acid

Ascorbic acid, or vitamin C, is a widely recognized antioxidant that plays a crucial protective role in the body (Conklin et al., 2024). Ascorbic acid directly scavenges superoxide anions, hydroxyl radicals, and other free radicals, and regenerates antioxidants such as vitamin E and GSH. It also modulates the expression of antioxidant enzymes like SOD and CAT, enhancing cellular antioxidant capacity and chelating metal ions to remove harmful ions such as iron and copper from the body (Chen et al., 2021). Systematic reviews indicate that ascorbic acid promotes neuronal differentiation of precursor cells, enhances adult hippocampal neurogenesis, and facilitates synaptic plasticity, thereby improving behavioral and biochemical changes in psychiatric disorders such as SZ, anxiety, MDD, and bipolar disorder (Moretti and Rodrigues, 2022). Evidence indicates that patients with SZ exhibit lower vitamin C levels (Myken et al., 2022). Research has shown that ascorbic acid can alleviate phenotypic symptoms of SZ by restoring the balance between ROS and antioxidant defenses (Dakhale et al., 2005; Damazio et al., 2017), reducing inflammatory factor levels, and employing other mechanisms (Supp et al., 2021). Similarly, ascorbic acid may exert antidepressant effects by restoring antioxidant enzyme activity (Moretti et al., 2013), activating the opioid receptor system (particularly the *μ*-opioid receptor), inhibiting NMDA receptors, or both (Moretti et al., 2018; Moretti et al., 2019). Furthermore, a recent study indicated that ascorbic acid can alleviate anxiety symptoms by upregulating synaptic proteins, increasing dendritic spine density, and promoting the maturation of the ventral dentate gyrus (DG) (Fraga et al., 2018; Fraga et al., 2020). Ascorbic acid can also directly enhance the catalytic activity of Tet methylcytosine dioxygenase 2 (TET2) in the oxidation of 5-methylcytosine (5mC), promote the folding and/or recycling of the cofactor Fe (2^+^) for TET2, and improve symptoms of depression (Ma et al., 2024; Yin et al., 2013).

These findings collectively highlight the therapeutic potential of ascorbic acid in treating mental illnesses.

### 3.6 Selenocompounds

Selenium is the active center of GPX, and recent advancements have led to the development of various mimetics designed to replicate GPX functions (Ferreira et al., 2021). The antioxidant mechanism of selenocompounds primarily involves the direct reaction of selenium atoms with free radicals generated by oxidative stress, thereby reducing the number of free radicals (Bartolini et al., 2017).

Serum selenium levels are considerably lower in patients with SZ compared to healthy controls (Li et al., 2018), suggesting a protective role for selenium in SZ and AN (Guo et al., 2023). Moreover, GPX activity is generally reported to be reduced by approximately 20% in patients with SZ. Supplementation with selenium has been shown to enhance cognitive function and improve clinical symptoms such as appetite and memory (Alsharif et al., 2023). Furthermore, dietary selenium appears to mitigate stress-induced depression symptoms, with epidemiological studies linking low selenium intake to an increased risk of severe depression (Pasco et al., 2012). However, this association has faced scrutiny from other studies (Guo et al., 2023; Bot et al., 2019). Animal studies have demonstrated the antidepressant and anti-anxiety properties of selenium compounds. For instance, F-DPS [2,5-diphenyl-3-(4-fluorobenzeneselenyl) selenophenyl] alleviates depression symptoms by restoring glutamate uptake in the PFC of mice (Gai et al., 2014a) and activating Extracellular Signal-Regulated Kinase (ERK) signaling (Gai et al., 2014b) pathways. MFSeI [1-methyl-3-(phenylselenyl)-1H indole] exerts antidepressant and anti-anxiety effects by reducing OS, regulating neurotransmitter balance, and affecting glucocorticoid receptor expression (Bampi et al., 2020). Diphenyl diselenide (DPDS) shows anti-anxiety effects by modulating Gamma-Aminobutyric Acid Type A (GABAA) and 5-Hydroxytryptamine (5HT) receptors (Ghisleni et al., 2008). Similarly, ebselen reduces impulsivity in rodent models and has been suggested as an alternative to lithium in the treatment of bipolar disorder and other mood disorders (Singh et al., 2016). Liquiritigenin display neuroprotection through exerting anti-oxidative and anti-inflammatory activities to suppress neuronal apoptosis (Chiu et al., 2018).

Selenium and its compounds show considerable potential in regulating nervous system functions, alleviating stress responses, and preventing mental illnesses. However, further research is necessary to confirm these findings.

### 3.7 Flavones

Flavonoids are low-molecular-weight compounds that belong to a class of plant secondary metabolites characterized by a polyphenolic structure. Flavonoids primarily exhibit their antioxidant mechanism by directly scavenging free radicals such as reactive oxygen species (ROS). Through specific functional groups, they react with free radicals to halt radical chain reactions. Moreover, flavonoids can also upregulate the activity of antioxidant enzymes, thereby enhancing the antioxidant defense system (Calis et al., 2020). They are categorized into six subcategories based on the carbon atoms connected to the C ring by the B ring, as well as the degree of unsaturation and oxidation of the C ring: flavanones, flavones, isoflavones, flavonols, chalcones, and anthocyanins (Hostetler et al., 2017).

Research has shown that 7,8-dihydroxyflavone (7,8-DHF) can alleviate SZ-like symptoms by effectively mimicing the effect of brain-derived neurotrophic factor (BDNF) in the brain (Jaehne et al., 2021) to selectively activate tyrosine kinase receptor B (TrkB) (Emili et al., 2022) and downstream Phospholipase C (PLC), AKT, and ERK1/2 signaling pathways. Similarly, the natural flavonoid 4′,5,7-trihydroxyflavone boosts the neurotrophic effects of BDNF by strengthening TrkB receptor signaling (Gao et al., 2023). Meanwhile, Deng et al. (2024) suggest that flavonoids have a protective role against depression, a finding supported by various animal and epidemiological studies (Amin et al., 2020; Zhang et al., 2015; Gui et al., 2023). Moreover, 7,8-DHF improves anxiety-like behavior in mice subjected to chronic alcohol exposure by regulating TrkB signaling in the amygdala (Wang et al., 2021). Natural flavonoids, such as chrysin, have demonstrated anxiolytic effects in animal models through mechanisms including interaction with the GABAA/benzodiazepine receptor complex and free radical scavenging (Karim et al., 2012; Gadotti and Zamponi, 2019; German-Ponciano et al., 2020). In summary, flavonoids possess significant therapeutic potential in treating SZ, depression, and AN due to their diverse biological activities and effects.

### 3.8 Zinc

Zinc, as an essential trace element, possesses the ability to modulate intracellular redox levels, preventing oxidative damage to biomembrane systems and reducing the formation of reactive oxygen species. Deficiency in zinc can increase the susceptibility of the body to oxidative stress, and appropriate supplementation can alleviate the resulting damage (Chasapis et al., 2020).

In the exploration of zinc’s potential in treating depression, a series of literature reviews have delved into the role of zinc in depression, including its potential mechanisms in regulating neurotransmitter, endocrine, and neurogenesis pathways, and have emphasized the reported antidepressant-like and mood-enhancing activities of zinc in both human and rodent intervention studies (Wang et al., 2019). Furthermore, a systematic review and meta-analysis found that zinc supplementation can alleviate depressive symptoms in patients undergoing antidepressant treatment (Da Silva et al., 2021). Another review has discussed the role of zinc in regulating brain-derived neurotrophic factor (BDNF) and its impact on neural function, suggesting that the combination of zinc supplementation with antidepressants can effectively treat major depressive disorder (Mlyniec, 2021). A preliminary study showed that individuals with anxiety have significantly elevated plasma copper levels and very low zinc levels, and supplementation with zinc significantly improved anxiety symptoms (Russo, 2011). However, the exact molecular mechanisms underlying the potential relevance of zinc have not been fully elucidated. Relevant animal studies have shown that zinc can regulate 5-HT receptors, exerting antidepressant effects (Satala et al., 2018). Additionally, zinc can block NMDA receptors, preventing glutamate from entering cells, thus producing an anxiolytic effect (Dou et al., 2018). It is noteworthy that the G protein-coupled receptor 39 (GPR39) is abundantly distributed in brain regions related to anxiety, and zinc, being a natural ligand for GPR39, is involved in the regulation of anxiety (Laitakari et al., 2021).These findings collectively highlight the therapeutic potential of zinc in treating mental illnesses.

### 3.9 Gut microbial biotransformation

Microbiota, particularly the gut microbiota, has been confirmed to play a significant role in neuropsychiatric health (Xiong et al., 2023). In the small intestine, the absorption of polyphenolic compounds is limited, hence the majority of these compounds reach the colon where they interact with the gut microbiota, exerting their oxidative activity (Ozdal et al., 2016; Wang et al., 2022).

Studies have shown that the gut microbiota can convert dietary polyphenols into low molecular weight bioactive metabolites, such as short-chain fatty acids (SCFAs) and phenolic acids, which may exert their antioxidant and anti-inflammatory effects through signaling pathways like Nrf2 and NF-κB (Balkrishna et al., 2024). Not only that, but polyphenols can utilize the structural characteristics of hydroxyl groups on their benzene rings to scavenge free radicals through H atom transfer (Papuc et al., 2017). Furthermore, polyphenols provide electrons to free radicals, stabilizing them and terminating the reaction. Epigallocatechin gallate (EGCG) from green tea can stimulate the nuclear translocation of Nrf2 in HepG2 cells, modulating the expression of antioxidant genes (Mi et al., 2018). Concurrently, polyphenolic compounds can exert neuroprotective effects by regulating adult neurogenesis, synaptogenesis, and neuroplasticity, as well as the activation of microglia (Godos et al., 2020). These studies provide in-depth insights into the relationship between gut microbiota and its metabolic components with mental health and offer directions for the development of dietary natural products for the prevention and treatment of psychiatric disorders.

## 4 Mechanisms underlying the effect of antioxidants on neuropsychiatric disorders

**Figure 2:**
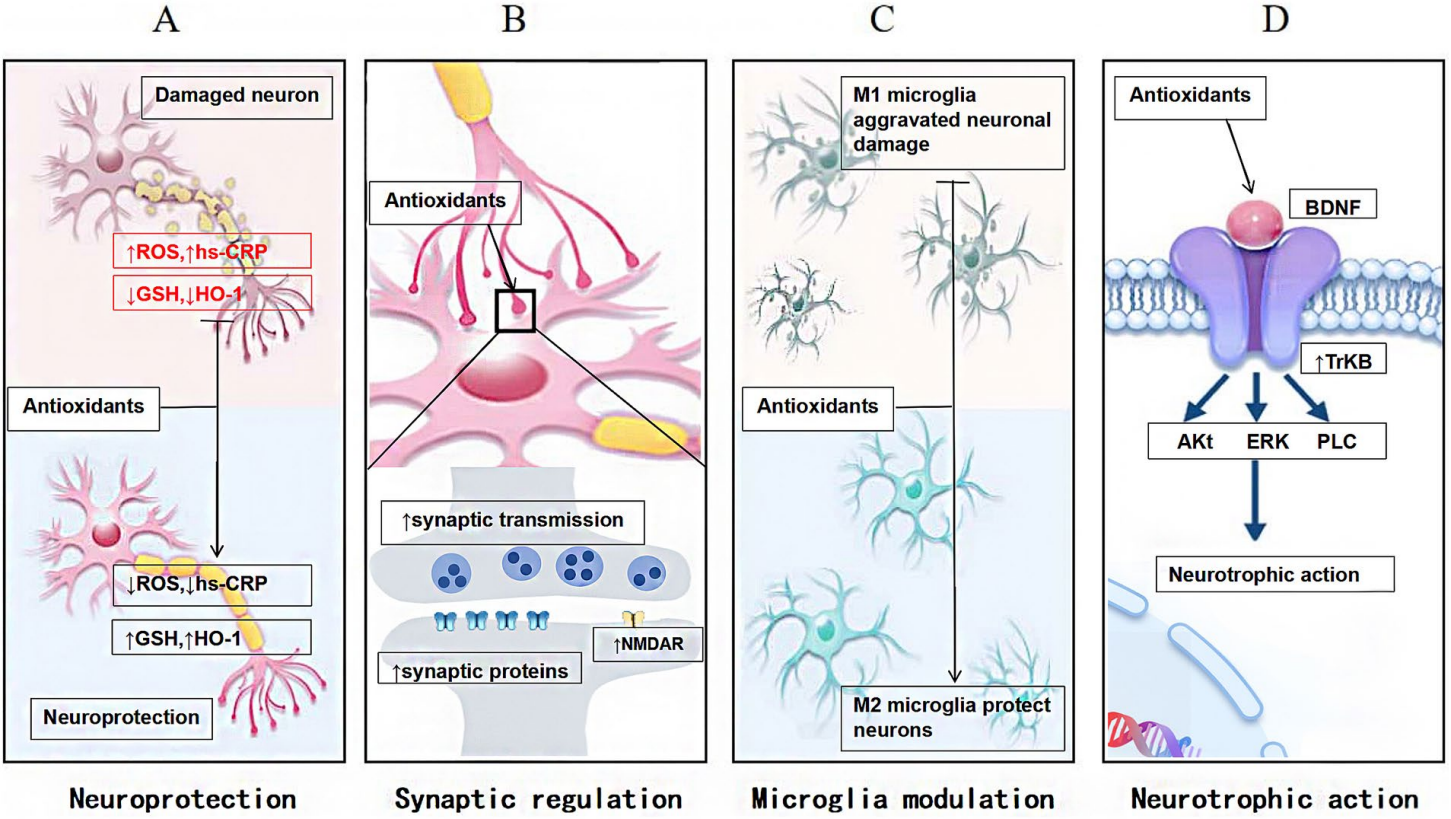
The mechanisms through which antioxidants impact neuropsychiatric disorders, including: **(A)** neuroprotection, which shields neurons from damage; **(B)** synaptic regulation, which modulates the transmission of signals between neurons; **(C)** modulation of microglial activity, which influences microglia polarization; and **(D)** neurotrophic effects, which support the growth and survival of neural cells.

The mechanisms through which antioxidants impact neuropsychiatric disorders can be summarized into several key areas: neuroprotection, synaptic regulation, modulation of microglial activity, and neurotrophic effects (as depicted in Figure 2).

### 4.1 Neuroprotection

Antioxidants are crucial in promoting the proliferation and differentiation of neural stem cells, enhancing neurons’ number and functionality, and ultimately improving functional recovery in the nervous system. For instance, SFN protects neurons from inflammation-mediated damage by lowering inflammatory markers such as high-sensitivity C-reactive protein (hs-CRP), restoring antioxidant enzyme expressions such as HO-1 and GSH, and reducing OS (Zeng et al., 2024). Similarly, ascorbic acid mitigates the production of ROS triggered by antipsychotic medications, thereby providing neuroprotective benefits (Dakhale et al., 2005; Damazio et al., 2017). Selenium supplementation can enhance the activity of GPX, thereby reducing OS damage to neurons (Bampi et al., 2020). Furthermore, flavonoids promote neuronal survival and repair through their antioxidant properties (Harvey, 2022).

### 4.2 Synaptic regulation

Antioxidants primarily function by mitigating OS effects on synaptic structure and function. Synaptic plasticity, characterized by dynamic synapse morphology, structure, and function changes, is essential for higher cognitive functions such as learning and memory (Magee and Grienberger, 2020). Antioxidants regulate synaptic plasticity by modulating neurons’ metabolic and signaling processes at pre- and postsynaptic levels.

For instance, NAC enhances synaptic transmission efficiency by promoting NMDA receptor activation and depolarizing the postsynaptic membrane (Phensy et al., 2017). This mechanism has been shown to alleviate long-term behavioral deficits associated with ketamine treatment in a preclinical SZ model during the perinatal period (Neill et al., 2022; Buhner et al., 2022). Selenium compounds can normalize glutamate uptake in the PFC, a process frequently disrupted in neuropsychiatric disorders (Gai et al., 2014a). Similarly, ascorbic acid can improve SZ symptoms by upregulating synaptic proteins, increasing dendritic spine density, and facilitating the maturation of ventral DG (Fraga et al., 2020).

### 4.3 Microglia modulation

Neuropsychiatric disorders are frequently characterized by increased OS and inflammatory responses, with abnormal activation and dysfunction of microglia playing a significant role (Lehmann et al., 2019). Antioxidants can slow the progression of these disorders by modulating microglial function and activity. For instance, SFN alleviates depressive symptoms by activating the Nrf2/HO-1 signaling, reducing microglial activation, and facilitating a transition to the M2 phenotype (Ferreira-Chamorro et al., 2018; He et al., 2022). Concurrently, NAC prevents behavioral deficits in mice by inhibiting microglial activation (Lehmann et al., 2019).

### 4.4 Neurotrophic action

Neurotrophic action refers to the effects of specific substances that promote neuron growth, development, maintenance, and regeneration (Castren and Monteggia, 2021). For instance, N-acetylcysteine ameliorates chemotherapy-induced impaired anxiety and depression-like behaviors by regulating BDNF release (Aslanlar et al., 2024). Furthermore, ALA can reverse ketamine-induced SZ-like symptoms in mice, potentially through its influence on BDNF in the PFC, as well as in a mouse model of depression (Vasconcelos et al., 2015; Aliomrani et al., 2022). Flavones enhance the neurotrophic effects of BDNF by reinforcing TrkB receptor signaling (Wang et al., 2021; Emili et al., 2022; Gao et al., 2023). Moreover, flavones significantly regulate neurotransmitter balance and improve the neuronal microenvironment, promoting neuronal nutrition and metabolic activity (Jaehne et al., 2021).

## 5 Conclusion

After a thorough review and analysis of existing literature, we have drawn the following conclusion: Antioxidants play a pivotal role in preventing neuropsychiatric disorders by effectively scavenging free radicals and mitigating oxidative stress, thereby forming a protective barrier for brain neural tissue. Specifically, antioxidants can efficiently neutralize reactive oxygen and nitrogen species, significantly reducing the damage these harmful molecules cause to brain neurons, and ensuring the preservation of neuronal structural and functional integrity. Furthermore, by regulating the synthesis, release, and reuptake of neurotransmitters, antioxidants maintain the normal functioning of the nervous system, providing robust support for the prevention of neuropsychiatric disorders. Additionally, antioxidants exhibit notable anti-inflammatory effects, inhibiting inflammatory responses and mitigating the damage caused by inflammatory mediators to neural tissue, thereby protecting the nervous system from inflammatory diseases. Lastly, antioxidants improve mitochondrial energy metabolism efficiency and antioxidant capacity, reducing the production of free radicals and further alleviating the potential damage caused by oxidative stress to neuronal cells.

While early studies suggest potential therapeutic effects of antioxidants in certain conditions, many of these studies are limited by small sample sizes, raising concerns about the reliability and reproducibility of the findings. Furthermore, the heterogeneity among patients remains a significant challenge in clinical trials. Factors such as physiological status, genetics, and lifestyle can significantly influence the effectiveness of antioxidant treatments. Moreover, the potential side effects of antioxidants may limit their therapeutic value. Therefore, a comprehensive evaluation of safety and efficacy is essential during drug development. Despite the numerous challenges and limitations associated with targeting OS for disease treatment, advances in science and technology, coupled with continued research, offer hope for overcoming these barriers. Future breakthroughs may provide novel approaches to disease prevention and treatment.

## References

[ref1] Al-Amin M. M. Sultana R. Sultana S. Rahman M. M. Reza H. M. (2016). Astaxanthin ameliorates prenatal LPS-exposed behavioral deficits and oxidative stress in adult offspring. BMC Neurosci. 17:11. doi: 10.1186/s12868-016-0245-z, PMID: 26856812 PMC4746928

[ref2] Aliomrani M. Mesripour A. Mehrjardi A. S. (2022). Creatine and alpha-lipoic acid antidepressant-like effect following cyclosporine a administration. Turk J Pharm Sci 19, 196–201. doi: 10.4274/tjps.galenos.2021.27217 35510329 PMC9083510

[ref3] Alle H. Roth A. Geiger J. R. (2009). Energy-efficient action potentials in hippocampal mossy fibers. Science 325, 1405–1408. doi: 10.1126/science.1174331 19745156

[ref4] Alsharif K. F. Albrakati A. Al Omairi N. E. Almalki A. S. Alsanie W. F. Elmageed Z. Y. A.. (2023). Therapeutic antischizophrenic activity of prodigiosin and selenium co-supplementation against amphetamine hydrochloride-induced behavioural changes and oxidative, inflammatory, and apoptotic challenges in rats. Environ. Sci. Pollut. Res. Int. 30, 7987–8001. doi: 10.1007/s11356-022-22409-x 36048389

[ref5] Amin N. Xie S. Tan X. Chen Y. Ren Q. Botchway B. O. A.. (2020). Optimized integration of fluoxetine and 7, 8-dihydroxyflavone as an efficient therapy for reversing depressive-like behavior in mice during the perimenopausal period. Prog. Neuro-Psychopharmacol. Biol. Psychiatry 101:109939. doi: 10.1016/j.pnpbp.2020.109939 32243998

[ref6] Andrade C. (2021). N-acetylcysteine augmentation for patients with major depressive disorder and bipolar depression. J. Clin. Psychiatry 82:891. doi: 10.4088/JCP.21f13891, PMID: 33999540

[ref7] Andreazza A. C. Cassini C. Rosa A. R. Almeida L. M. Nardin P. Cunha A. B. N.. (2007). Serum S100B and antioxidant enzymes in bipolar patients. J. Psychiatr. Res. 41, 523–529. doi: 10.1016/j.jpsychires.2006.07.013 16956621

[ref8] Aslanlar D. A. Visneci E. F. Oz M. Nurullahoglu Atalik K. E. (2024). N-acetylcysteine ameliorates chemotherapy-induced impaired anxiety and depression-like behaviors by regulating inflammation, oxidative and cholinergic status, and BDNF release. Behav. Brain Res. 458:114740. doi: 10.1016/j.bbr.2023.114740 37926333

[ref9] Bader M. Abdelwanis M. Maalouf M. Jelinek H. F. (2024). Detecting depression severity using weighted random forest and oxidative stress biomarkers. Sci. Rep. 14:16328. doi: 10.1038/s41598-024-67251-y, PMID: 39009760 PMC11250802

[ref10] Balkrishna A. Verma S. Singh S. K. Dobhal V. Arya V. (2024). Exploring the antioxidant mechanisms of millet polyphenols: regulation of Nrf2 and NF-κB and their impact on gut microbiota. Discov. Food 4:199. doi: 10.1007/s44187-024-00199-0

[ref11] Bampi S. R. Casaril A. M. Fronza M. G. Domingues M. Vieira B. Begnini K. R.. (2020). The selenocompound 1-methyl-3-(phenylselanyl)-1H-indole attenuates depression-like behavior, oxidative stress, and neuroinflammation in streptozotocin-treated mice. Brain Res. Bull. 161, 158–165. doi: 10.1016/j.brainresbull.2020.05.008 32470357

[ref9021] Bartolini D. Sancineto L. de Bem A. F. Tew K. D. Santi C. Radi R.. (2017). Selenocompounds in Cancer Therapy: An overview. Adv. Cancer Res. 136, 259–302. doi: 10.1016/bs.acr.2017.07.007 29054421

[ref12] Bellisario V. Berry A. Capoccia S. Raggi C. Panetta P. Branchi I.. (2014). Gender-dependent resiliency to stressful and metabolic challenges following prenatal exposure to high-fat diet in the p66(Shc‑/‑) mouse. Front. Behav. Neurosci. 8:285. doi: 10.3389/fnbeh.2014.00285, PMID: 25202246 PMC4141279

[ref13] Bersuker K. Hendricks J. M. Li Z. Magtanong L. Ford B. Tang P. H.. (2019). The CoQ oxidoreductase FSP1 acts parallel to GPX4 to inhibit ferroptosis. Nature 575, 688–692. doi: 10.1038/s41586-019-1705-2, PMID: 31634900 PMC6883167

[ref14] Block M. L. Zecca L. Hong J. S. (2007). Microglia-mediated neurotoxicity: uncovering the molecular mechanisms. Nat. Rev. Neurosci. 8, 57–69. doi: 10.1038/nrn2038 17180163

[ref15] Bot M. Brouwer I. A. Roca M. Kohls E. Penninx B. Watkins E.. (2019). Effect of multinutrient supplementation and food-related behavioral activation therapy on prevention of major depressive disorder among overweight or obese adults with Subsyndromal depressive symptoms: the MooDFOOD randomized clinical trial. JAMA 321, 858–868. doi: 10.1001/jama.2019.0556 30835307 PMC6439597

[ref16] Bruno A. Pandolfo G. Crucitti M. Lorusso S. Zoccali R. A. Muscatello M. R. (2016). Acetyl-L-carnitine augmentation of clozapine in partial-responder schizophrenia: a 12-week, open-label uncontrolled preliminary study. Clin. Neuropharmacol. 39, 277–280. doi: 10.1097/WNF.0000000000000170, PMID: 27404738

[ref17] Buhner L. M. Kapanaiah S. K. T. Katzel D. (2022). Chronic N-acetylcysteine treatment improves anhedonia and cognition in a mouse model of the schizophrenia prodrome. Front. Behav. Neurosci. 16:1002223. doi: 10.3389/fnbeh.2022.1002223 36225391 PMC9548602

[ref18] Calis Z. Mogulkoc R. Baltaci A. K. (2020). The roles of Flavonols/flavonoids in neurodegeneration and Neuroinflammation. Mini Rev. Med. Chem. 20, 1475–1488. doi: 10.2174/1389557519666190617150051, PMID: 31288717

[ref19] Cao B. Wang D. Pan Z. Brietzke E. McIntyre R. S. Musial N.. (2019). Characterizing acyl-carnitine biosignatures for schizophrenia: a longitudinal pre- and post-treatment study. Transl. Psychiatry 9:19. doi: 10.1038/s41398-018-0353-x, PMID: 30655505 PMC6336814

[ref20] Cao B. Wang D. Pan Z. McIntyre R. S. Brietzke E. Subramanieapillai M.. (2020). Metabolic profiling for water-soluble metabolites in patients with schizophrenia and healthy controls in a Chinese population: a case-control study. World J. Biol. Psychiatry 21, 357–367. doi: 10.1080/15622975.2019.1615639, PMID: 31161852

[ref21] Castren E. Monteggia L. M. (2021). Brain-derived neurotrophic factor signaling in depression and antidepressant action. Biol. Psychiatry 90, 128–136. doi: 10.1016/j.biopsych.2021.05.008 34053675

[ref22] Chasapis C. T. Ntoupa P. A. Spiliopoulou C. A. Stefanidou M. E. (2020). Recent aspects of the effects of zinc on human health. Arch. Toxicol. 94, 1443–1460. doi: 10.1007/s00204-020-02702-9, PMID: 32394086

[ref23] Chen D. Ai X. Li Y. Li Y. Ao Y. Rong J.. (2023). Protective effects of cu/Zn-SOD and Mn-SOD on UVC radiation-induced damage in NIH/3T3 cells and murine skin. Acta Histochem. 125:152030. doi: 10.1016/j.acthis.2023.152030, PMID: 37099996

[ref24] Chen L. Wang W. Zhang J. Cui H. Ni D. Jiang H. (2021). Dual effects of ascorbic acid on the stability of EGCG by the oxidation product dehydroascorbic acid promoting the oxidation and inhibiting the hydrolysis pathway. Food Chem. 337:127639. doi: 10.1016/j.foodchem.2020.127639, PMID: 32799157

[ref25] Chien Y. L. Hwu H. G. Hwang T. J. Hsieh M. H. Liu C. C. Lin-Shiau S. Y.. (2020). Clinical implications of oxidative stress in schizophrenia: acute relapse and chronic stable phase. Prog. Neuro-Psychopharmacol. Biol. Psychiatry 99:109868. doi: 10.1016/j.pnpbp.2020.109868 31954755

[ref26] Chiu Y. J. Lee C. M. Lin T. H. Lin H. Y. Lee S. Y. Mesri M.. (2018). Chinese herbal medicine Glycyrrhiza inflataReduces Abeta aggregation and exerts neuroprotection through anti-oxidation and anti-inflammation. Am. J. Chin. Med. 1, 1–25. doi: 10.1142/S0192415X18500799 30284464

[ref27] Chung C. P. Schmidt D. Stein C. M. Morrow J. D. Salomon R. M. (2013). Increased oxidative stress in patients with depression and its relationship to treatment. Psychiatry Res. 206, 213–216. doi: 10.1016/j.psychres.2012.10.018, PMID: 23245537 PMC3615036

[ref28] Cobley J. N. Fiorello M. L. Bailey D. M. (2018). 13 reasons why the brain is susceptible to oxidative stress. Redox Biol. 15, 490–503. doi: 10.1016/j.redox.2018.01.008, PMID: 29413961 PMC5881419

[ref29] Conklin P. L. Foyer C. H. Hancock R. D. Ishikawa T. Smirnoff N. (2024). Ascorbic acid metabolism and functions. J. Exp. Bot. 75, 2599–2603. doi: 10.1093/jxb/erae143, PMID: 38699987 PMC11066792

[ref30] Da Silva L. E. de Oliveira M. P. da Silva M. R. Abel J. D. S. Tartari G. de Aguiar M.. (2023). L-carnitine and acetyl-L carnitine: a possibility for treating alterations induced by obesity in the central nervous system. Neurochem. Res. 48, 3316–3326. doi: 10.1007/s11064-023-04000-z 37495838

[ref31] da Silva L. E. M. de Santana M. L. P. Costa P. R. F. Pereira E. M. Nepomuceno C. M. M. Queiroz V. A. O.. (2021). Zinc supplementation combined with antidepressant drugs for treatment of patients with depression: a systematic review and meta-analysis. Nutr. Rev. 79, 1–12. doi: 10.1093/nutrit/nuaa039, PMID: 32885249

[ref32] Dakhale G. N. Khanzode S. D. Khanzode S. S. Saoji A. (2005). Supplementation of vitamin C with atypical antipsychotics reduces oxidative stress and improves the outcome of schizophrenia. Psychopharmacology 182, 494–498. doi: 10.1007/s00213-005-0117-1, PMID: 16133138

[ref33] Damazio L. S. Silveira F. R. Canever L. Castro A. A. Estrela J. M. Budni J.. (2017). The preventive effects of ascorbic acid supplementation on locomotor and acetylcholinesterase activity in an animal model of schizophrenia induced by ketamine. An. Acad. Bras. Cienc. 89, 1133–1141. doi: 10.1590/0001-3765201720160490, PMID: 28513779

[ref34] Dang R. Wang M. Li X. Wang H. Liu L. Wu Q.. (2022). Edaravone ameliorates depressive and anxiety-like behaviors via Sirt1/Nrf2/HO-1/Gpx4 pathway. J. Neuroinflammation 19:41. doi: 10.1186/s12974-022-02400-6, PMID: 35130906 PMC8822843

[ref35] De Lima D. N. Jr. Filho C. W. L. C. Frota I. J. de Oliveira A. L. B. Menezes C. E. S. Filho A. J. M. C.. (2023). Alpha-lipoic acid as adjunctive treatment for schizophrenia: a randomized double-blind study. J. Clin. Psychopharmacol. 43, 39–45. doi: 10.1097/JCP.0000000000001639, PMID: 36584248

[ref36] Deng M. G. Liu F. Wang K. Zhang M. J. Feng Q. Liu J. (2024). Association between dietary flavonoid intake and depressive symptoms: a cross-sectional research. Gen. Hosp. Psychiatry 86, 75–84. doi: 10.1016/j.genhosppsych.2023.12.005, PMID: 38134552

[ref37] Dickerson F. Origoni A. Katsafanas E. Squire A. Newman T. Fahey J.. (2021). Randomized controlled trial of an adjunctive sulforaphane nutraceutical in schizophrenia. Schizophr. Res. 231, 142–144. doi: 10.1016/j.schres.2021.03.018, PMID: 33839372

[ref38] Dickinson B. C. Peltier J. Stone D. Schaffer D. V. Chang C. J. (2011). Nox2 redox signaling maintains essential cell populations in the brain. Nat. Chem. Biol. 7, 106–112. doi: 10.1038/nchembio.497, PMID: 21186346 PMC3023843

[ref39] Ding Q. Tian Y. Wang X. Li P. Su D. Wu C.. (2020). Oxidative damage of tryptophan Hydroxylase-2 mediated by Peroxisomal superoxide anion radical in brains of mouse with depression. J. Am. Chem. Soc. 142, 20735–20743. doi: 10.1021/jacs.0c09576, PMID: 33237755

[ref40] Distler M. G. Plant L. D. Sokoloff G. Hawk A. J. Aneas I. Wuenschell G. E.. (2012). Glyoxalase 1 increases anxiety by reducing GABAA receptor agonist methylglyoxal. J. Clin. Invest. 122, 2306–2315. doi: 10.1172/JCI61319, PMID: 22585572 PMC3366407

[ref41] Dou M. Gong A. Liang H. Wang Q. Wu Y. Ma A.. (2018). Improvement of symptoms in a rat model of depression through combined zinc and folic acid administration via up-regulation of the Trk B and NMDA. Neurosci. Lett. 683, 196–201. doi: 10.1016/j.neulet.2018.07.036 30056106

[ref42] Dwir D. Giangreco B. Xin L. Tenenbaum L. Cabungcal J. H. Steullet P.. (2020). Correction: MMP9/RAGE pathway overactivation mediates redox dysregulation and neuroinflammation, leading to inhibitory/excitatory imbalance: a reverse translation study in schizophrenia patients. Mol. Psychiatry 25:3105. doi: 10.1038/s41380-020-0716-6, PMID: 32218526 PMC7962564

[ref43] Ebrahimi M. Ahangar N. Zamani E. Shaki F. (2023). L-carnitine prevents Behavioural alterations in ketamine-induced schizophrenia in mice: possible involvement of oxidative stress and inflammation pathways. J Toxicol 2023:9093231. doi: 10.1155/2023/9093231 37363159 PMC10289879

[ref44] El-Houseiny W. Arisha A. H. Metwally M. M. M. Abdel-Warith A. A. Younis E. M. Davies S. J.. (2023). Alpha-lipoic acid suppresses gibberellic acid nephrotoxicity in Nile tilapia (*Oreochromis niloticus*) via modulating oxidative stress, inflammation, cytokine production, and apoptosis. Pestic. Biochem. Physiol. 196:105598. doi: 10.1016/j.pestbp.2023.105598, PMID: 37945227

[ref45] El-Shoura E. A. M. Abdelzaher L. A. Mahmoud N. I. Farghaly O. A. Sabry M. Girgis Shahataa M.. (2024). Combined sulforaphane and beta-sitosterol mitigate olanzapine-induced metabolic disorders in rats: insights on FOXO, PI3K/AKT, JAK/STAT3, and MAPK signaling pathways. Int. Immunopharmacol. 140:112904. doi: 10.1016/j.intimp.2024.112904, PMID: 39116489

[ref46] Emili M. Guidi S. Uguagliati B. Giacomini A. Bartesaghi R. Stagni F. (2022). Treatment with the flavonoid 7,8-Dihydroxyflavone: a promising strategy for a constellation of body and brain disorders. Crit. Rev. Food Sci. Nutr. 62, 13–50. doi: 10.1080/10408398.2020.1810625, PMID: 32914634

[ref47] Emsley R. Chiliza B. Asmal L. du Plessis S. Phahladira L. van Niekerk E.. (2014). A randomized, controlled trial of omega-3 fatty acids plus an antioxidant for relapse prevention after antipsychotic discontinuation in first-episode schizophrenia. Schizophr. Res. 158, 230–235. doi: 10.1016/j.schres.2014.06.004, PMID: 24996507

[ref48] Ferreira R. L. U. Sena-Evangelista K. C. M. de Azevedo E. P. Pinheiro F. I. Cobucci R. N. Pedrosa L. F. C. (2021). Selenium in human health and gut microflora: bioavailability of Selenocompounds and relationship with diseases. Front. Nutr. 8:685317. doi: 10.3389/fnut.2021.685317, PMID: 34150830 PMC8211732

[ref49] Ferreira-Chamorro P. Redondo A. Riego G. Leanez S. Pol O. (2018). Sulforaphane inhibited the nociceptive responses, anxiety- and depressive-like behaviors associated with neuropathic pain and improved the anti-allodynic effects of morphine in mice. Front. Pharmacol. 9:1332. doi: 10.3389/fphar.2018.01332, PMID: 30542282 PMC6277937

[ref50] Filiou M. D. Sandi C. (2019). Anxiety and brain mitochondria: a bidirectional crosstalk. Trends Neurosci. 42, 573–588. doi: 10.1016/j.tins.2019.07.002, PMID: 31362874

[ref51] Fizikova I. Dragasek J. Racay P. (2023). Mitochondrial dysfunction, altered mitochondrial oxygen, and energy metabolism associated with the pathogenesis of schizophrenia. Int. J. Mol. Sci. 24:697. doi: 10.3390/ijms24097991, PMID: 37175697 PMC10178941

[ref52] Fornaro M. Caiazza C. Billeci M. Berk M. Marx W. Balanza-Martinez V.. (2024). Nutraceuticals and phytoceuticals in the treatment of schizophrenia: a systematic review and network meta-analysis "Nutra NMA SCZ". Mol. Psychiatry 2024:2645. doi: 10.1038/s41380-024-02645-y, PMID: 39026098

[ref53] Fraga D. B. Costa A. P. Olescowicz G. Camargo A. Pazini F. L. Freitas A. E.. (2020). Ascorbic acid presents rapid behavioral and hippocampal synaptic plasticity effects. Prog. Neuro-Psychopharmacol. Biol. Psychiatry 96:109757. doi: 10.1016/j.pnpbp.2019.109757, PMID: 31476335

[ref54] Fraga D. B. Olescowicz G. Moretti M. Siteneski A. Tavares M. K. Azevedo D.. (2018). Anxiolytic effects of ascorbic acid and ketamine in mice. J. Psychiatr. Res. 100, 16–23. doi: 10.1016/j.jpsychires.2018.02.006 29475017

[ref55] Gadotti V. M. Zamponi G. W. (2019). Anxiolytic effects of the flavonoid luteolin in a mouse model of acute colitis. Mol. Brain 12:114. doi: 10.1186/s13041-019-0539-z, PMID: 31878979 PMC6933648

[ref56] Gai B. M. Bortolatto C. F. Heck S. O. Stein A. L. Duarte M. M. Zeni G.. (2014a). An organoselenium compound improves behavioral, endocrinal and neurochemical changes induced by corticosterone in mice. Psychopharmacology 231, 2119–2130. doi: 10.1007/s00213-013-3361-9, PMID: 24306280

[ref57] Gai B. M. Sanna M. D. Stein A. L. Zeni G. Galeotti N. Nogueira C. W. (2014b). ERK1/2 phosphorylation is involved in the antidepressant-like action of 2,5-diphenyl-3-(4-fluorophenylseleno)-selenophene in mice. Eur. J. Pharmacol. 736, 44–54. doi: 10.1016/j.ejphar.2014.04.033, PMID: 24797783

[ref58] Gao A. X. Xia T. C. Lin L. S. Dong T. T. Tsim K. W. (2023). The neurotrophic activities of brain-derived neurotrophic factor are potentiated by binding with apigenin, a common flavone in vegetables, in stimulating the receptor signaling. CNS Neurosci. Ther. 29, 2787–2799. doi: 10.1111/cns.14230, PMID: 37101380 PMC10493664

[ref59] Garcia-Gutierrez M. S. Navarrete F. Sala F. Gasparyan A. Austrich-Olivares A. Manzanares J. (2020). Biomarkers in psychiatry: concept, definition, types and relevance to the clinical reality. Front. Psych. 11:432. doi: 10.3389/fpsyt.2020.00432, PMID: 32499729 PMC7243207

[ref60] Geng Y. Zhang H. Zhang G. Zhou J. Zhu M. Ma L.. (2023). Near-infrared fluorescent probe for the in situ visualization of oxidative stress in the brains of Neuroinflammatory and schizophrenic mice. Anal. Chem. 95, 11943–11952. doi: 10.1021/acs.analchem.3c01447 37526416 PMC10433243

[ref61] German-Ponciano L. J. Dutra Costa B. P. Feitosa L. M. Campos K. D. S. da Silva Chaves S. N. Cueto-Escobedo J.. (2020). Chrysin, but not flavone backbone, decreases anxiety-like behavior in animal screens. Neurochem. Int. 140:104850. doi: 10.1016/j.neuint.2020.104850 32961254

[ref62] Ghisleni G. Kazlauckas V. Both F. L. Pagnussat N. Mioranzza S. Rocha J. B.. (2008). Diphenyl diselenide exerts anxiolytic-like effect in Wistar rats: putative roles of GABAA and 5HT receptors. Prog. Neuro-Psychopharmacol. Biol. Psychiatry 32, 1508–1515. doi: 10.1016/j.pnpbp.2008.05.008, PMID: 18579279

[ref63] Godos J. Currenti W. Angelino D. Mena P. Castellano S. Caraci F.. (2020). Diet and mental health: review of the recent updates on molecular mechanisms. Antioxidants (Basel) 9:346. doi: 10.3390/antiox9040346 32340112 PMC7222344

[ref64] Goh X. X. Tang P. Y. Tee S. F. (2021). 8-Hydroxy-2'-Deoxyguanosine and reactive oxygen species as biomarkers of oxidative stress in mental illnesses: a Meta-analysis. Psychiatry Investig. 18, 603–618. doi: 10.30773/pi.2020.0417, PMID: 34340273 PMC8328836

[ref65] Goh X. X. Tang P. Y. Tee S. F. (2022). Effects of antipsychotics on antioxidant defence system in patients with schizophrenia: a meta-analysis. Psychiatry Res. 309:114429. doi: 10.1016/j.psychres.2022.114429 35150976

[ref66] Gui J. Han Z. Ding R. Yang X. Yang J. Luo H.. (2023). Depression associated with dietary intake of flavonoids: an analysis of data from the National Health and nutrition examination survey, 2007-2010. J. Psychosom. Res. 173:111468. doi: 10.1016/j.jpsychores.2023.111468, PMID: 37611347

[ref67] Guo X. Tang P. Hou C. Li R. (2023). Mendelian randomization investigation highlights different roles of selenium status in mental disorders. Prog. Neuro-Psychopharmacol. Biol. Psychiatry 122:110694. doi: 10.1016/j.pnpbp.2022.110694, PMID: 36521586

[ref68] Han Q. Q. Wu P. F. Li Y. H. Cao Y. Chen J. G. Wang F. (2022). SVCT2-mediated ascorbic acid uptake buffers stress responses via DNA hydroxymethylation reprogramming of S100 calcium-binding protein A4 gene. Redox Biol. 58:102543. doi: 10.1016/j.redox.2022.102543, PMID: 36436457 PMC9694147

[ref69] Harvey J. (2022). Food for thought: leptin and hippocampal synaptic function. Front. Pharmacol. 13:882158. doi: 10.3389/fphar.2022.882158, PMID: 35784728 PMC9247348

[ref70] He L. Zheng Y. Huang L. Ye J. Ye Y. Luo H.. (2022). Nrf2 regulates the arginase 1(+) microglia phenotype through the initiation of TREM2 transcription, ameliorating depression-like behavior in mice. Transl. Psychiatry 12:459. doi: 10.1038/s41398-022-02227-y, PMID: 36316319 PMC9622811

[ref71] Hoepner C. T. McIntyre R. S. Papakostas G. I. (2021). Impact of supplementation and nutritional interventions on pathogenic processes of mood disorders: a review of the evidence. Nutrients 13:767. doi: 10.3390/nu13030767, PMID: 33652997 PMC7996954

[ref72] Holmquist L. Stuchbury G. Berbaum K. Muscat S. Young S. Hager K.. (2007). Lipoic acid as a novel treatment for Alzheimer's disease and related dementias. Pharmacol. Ther. 113, 154–164. doi: 10.1016/j.pharmthera.2006.07.001 16989905

[ref73] Hostetler G. L. Ralston R. A. Schwartz S. J. (2017). Flavones: food sources, bioavailability, metabolism, and bioactivity. Adv. Nutr. 8, 423–435. doi: 10.3945/an.116.012948, PMID: 28507008 PMC5421117

[ref74] Hou X. Y. Hu Z. L. Zhang D. Z. Lu W. Zhou J. Wu P. F.. (2017). Rapid antidepressant effect of hydrogen sulfide: evidence for activation of mTORC1-TrkB-AMPA receptor pathways. Antioxid. Redox Signal. 27, 472–488. doi: 10.1089/ars.2016.6737, PMID: 28158955

[ref75] Hovatta I. Tennant R. S. Helton R. Marr R. A. Singer O. Redwine J. M.. (2005). Glyoxalase 1 and glutathione reductase 1 regulate anxiety in mice. Nature 438, 662–666. doi: 10.1038/nature04250, PMID: 16244648

[ref76] Iannuzzo F. Basile G. A. Campolo D. Genovese G. Pandolfo G. Giunta L.. (2022). Metabolic and clinical effect of alpha-lipoic acid administration in schizophrenic subjects stabilized with atypical antipsychotics: a 12-week, open-label, uncontrolled study. Curr Res Pharmacol Drug Discov 3:100116. doi: 10.1016/j.crphar.2022.100116, PMID: 35992380 PMC9389248

[ref77] Jaehne E. J. Chong E. M. S. Sbisa A. Gillespie B. Hill R. Gogos A.. (2021). TrkB agonist 7,8-dihydroxyflavone reverses an induced prepulse inhibition deficit selectively in maternal immune activation offspring: implications for schizophrenia. Behav. Pharmacol. 32, 404–412. doi: 10.1097/FBP.0000000000000632, PMID: 33883449

[ref78] Jia R. Yuan X. Zhang X. Song P. Han S. Wang S.. (2023). Oxidative stress impairs cognitive function by affecting hippocampal fimbria volume in drug-naive, first-episode schizophrenia. Front. Neurosci. 17:1153439. doi: 10.3389/fnins.2023.1153439 37139526 PMC10149877

[ref79] Jimenez-Fernandez S. Gurpegui M. Garrote-Rojas D. Gutierrez-Rojas L. Carretero M. D. Correll C. U. (2022). Oxidative stress parameters and antioxidants in adults with unipolar or bipolar depression versus healthy controls: systematic review and meta-analysis. J. Affect. Disord. 314, 211–221. doi: 10.1016/j.jad.2022.07.015, PMID: 35868596

[ref80] Kamal M. M. Akter S. Lin C. N. Nazzal S. (2020). Sulforaphane as an anticancer molecule: mechanisms of action, synergistic effects, enhancement of drug safety, and delivery systems. Arch. Pharm. Res. 43, 371–384. doi: 10.1007/s12272-020-01225-2, PMID: 32152852

[ref81] Kargar H. M. P. Noshiri H. (2024). Protective effects of alpha-lipoic acid on anxiety-like behavior, memory and prevention of hippocampal oxidative stress in methamphetamine-treated rats. Psychopharmacology 241, 315–326. doi: 10.1007/s00213-023-06487-4, PMID: 37882813

[ref82] Karim N. Curmi J. Gavande N. Johnston G. A. Hanrahan J. R. Tierney M. L.. (2012). 2′-Methoxy-6-methylflavone: a novel anxiolytic and sedative with subtype selective activating and modulating actions at GABA(a) receptors. Br. J. Pharmacol. 165, 880–896. doi: 10.1111/j.1476-5381.2011.01604.x, PMID: 21797842 PMC3312486

[ref84] Kaya M. C. Bez Y. Karababa I. F. Emhan A. Aksoy N. Bulut M.. (2013). Decreased serum sulphydryl levels as a sign of increased oxidative stress in generalized anxiety disorder. Psychiatry Investig. 10, 281–285. doi: 10.4306/pi.2013.10.3.281 PMC3843021 24302952

[ref85] Kirkpatrick R. H. Munoz D. P. Khalid-Khan S. Booij L. (2021). Methodological and clinical challenges associated with biomarkers for psychiatric disease: a scoping review. J. Psychiatr. Res. 143, 572–579. doi: 10.1016/j.jpsychires.2020.11.023 33221025

[ref86] Kiser C. Gonul C. P. Olcum M. Genc S. (2021). Inhibitory effects of sulforaphane on NLRP3 inflammasome activation. Mol. Immunol. 140, 175–185. doi: 10.1016/j.molimm.2021.10.014 34717147

[ref87] Koskinen M. K. Hovatta I. (2023). Genetic insights into the neurobiology of anxiety. Trends Neurosci. 46, 318–331. doi: 10.1016/j.tins.2023.01.007, PMID: 36828693

[ref88] Kotan V. O. Sarandol E. Kirhan E. Ozkaya G. Kirli S. (2011). Effects of long-term antidepressant treatment on oxidative status in major depressive disorder: a 24-week follow-up study. Prog. Neuro-Psychopharmacol. Biol. Psychiatry 35, 1284–1290. doi: 10.1016/j.pnpbp.2011.03.021, PMID: 21515329

[ref89] Kulak A. Steullet P. Cabungcal J. H. Werge T. Ingason A. Cuenod M.. (2013). Redox dysregulation in the pathophysiology of schizophrenia and bipolar disorder: insights from animal models. Antioxid. Redox Signal. 18, 1428–1443. doi: 10.1089/ars.2012.4858, PMID: 22938092

[ref90] Laitakari A. Liu L. Frimurer T. M. Holst B. (2021). The zinc-sensing receptor GPR39 in physiology and as a pharmacological target. Int. J. Mol. Sci. 22:872. doi: 10.3390/ijms22083872, PMID: 33918078 PMC8070507

[ref91] Lehmann M. L. Weigel T. K. Poffenberger C. N. Herkenham M. (2019). The behavioral sequelae of social defeat require microglia and are driven by oxidative stress in mice. J. Neurosci. 39, 5594–5605. doi: 10.1523/JNEUROSCI.0184-19.2019, PMID: 31085604 PMC6616288

[ref92] Lennicke C. Cocheme H. M. (2021). Redox metabolism: ROS as specific molecular regulators of cell signaling and function. Mol. Cell 81, 3691–3707. doi: 10.1016/j.molcel.2021.08.018 34547234

[ref93] Li S. Jiang J. Zhu W. Wang D. Dong C. Bu Y.. (2024). Increased cell-free DNA is associated with oxidative damage in patients with schizophrenia. J. Psychiatr. Res. 175, 20–28. doi: 10.1016/j.jpsychires.2024.04.047 38701608

[ref94] Li Z. Liu Y. Li X. Ju W. Wu G. Yang X.. (2018). Association of Elements with schizophrenia and intervention of selenium supplements. Biol. Trace Elem. Res. 183, 16–21. doi: 10.1007/s12011-017-1105-0, PMID: 28812245

[ref95] Liang L. Chen J. Xiao L. Wang Q. Wang G. (2022). Mitochondrial modulators in the treatment of bipolar depression: a systematic review and meta-analysis. Transl. Psychiatry 12:4. doi: 10.1038/s41398-021-01727-7, PMID: 35013098 PMC8748981

[ref96] Lolak S. Suwannarat P. Lipsky R. H. (2014). Epigenetics of depression. Prog. Mol. Biol. Transl. Sci. 128, 103–137. doi: 10.1016/B978-0-12-800977-2.00005-X 25410543

[ref97] Ma C. Gu C. Lian P. Wazir J. Lu R. Ruan B.. (2023). Sulforaphane alleviates psoriasis by enhancing antioxidant defense through KEAP1-NRF2 pathway activation and attenuating inflammatory signaling. Cell Death Dis. 14:768. doi: 10.1038/s41419-023-06234-9, PMID: 38007430 PMC10676357

[ref98] Ma X. Xu S. Zhou Y. Zhang Q. Yang H. Wan B.. (2024). Targeting Nr2e3 to modulate Tet2 expression: therapeutic potential for depression treatment. Adv Sci (Weinh) 11:e2400726. doi: 10.1002/advs.202400726, PMID: 38881534 PMC11336902

[ref99] Maas D. A. Eijsink V. D. van Hulten J. A. Panic R. De Weerd P. Homberg J. R.. (2021). Antioxidant treatment ameliorates prefrontal hypomyelination and cognitive deficits in a rat model of schizophrenia. Neuropsychopharmacology 46, 1161–1171. doi: 10.1038/s41386-021-00964-0, PMID: 33564104 PMC8115238

[ref100] Maas D. A. Valles A. Martens G. J. M. (2017). Oxidative stress, prefrontal cortex hypomyelination and cognitive symptoms in schizophrenia. Transl. Psychiatry 7:e1171. doi: 10.1038/tp.2017.138, PMID: 28934193 PMC5538118

[ref101] MacDowell K. S. Caso J. R. Martin-Hernandez D. Moreno B. M. Madrigal J. L. M. Mico J. A.. (2016). The atypical antipsychotic Paliperidone regulates endogenous antioxidant/anti-inflammatory pathways in rat models of acute and chronic restraint stress. Neurotherapeutics 13, 833–843. doi: 10.1007/s13311-016-0438-2, PMID: 27233514 PMC5081131

[ref102] Maes M. Landucci Bonifacio K. Morelli N. R. Vargas H. O. Barbosa D. S. Carvalho A. F.. (2019). Major differences in Neurooxidative and Neuronitrosative stress pathways between major depressive disorder and types I and II bipolar disorder. Mol. Neurobiol. 56, 141–156. doi: 10.1007/s12035-018-1051-7, PMID: 29681025

[ref103] Magee J. C. Grienberger C. (2020). Synaptic plasticity forms and functions. Annu. Rev. Neurosci. 43, 95–117. doi: 10.1146/annurev-neuro-090919-022842 32075520

[ref104] Magistretti P. J. Allaman I. (2015). A cellular perspective on brain energy metabolism and functional imaging. Neuron 86, 883–901. doi: 10.1016/j.neuron.2015.03.035, PMID: 25996133

[ref105] Maiorino M. Conrad M. Ursini F. (2018). GPx4, lipid peroxidation, and cell death: discoveries, rediscoveries, and open issues. Antioxid. Redox Signal. 29, 61–74. doi: 10.1089/ars.2017.7115, PMID: 28462584

[ref106] Mangla B. Javed S. Sultan M. H. Kumar P. Kohli K. Najmi A.. (2021). Sulforaphane: a review of its therapeutic potentials, advances in its nanodelivery, recent patents, and clinical trials. Phytother. Res. 35, 5440–5458. doi: 10.1002/ptr.7176 34184327

[ref107] Mi Y. Zhang W. Tian H. Li R. Huang S. Li X.. (2018). EGCG evokes Nrf2 nuclear translocation and dampens PTP1B expression to ameliorate metabolic misalignment under insulin resistance condition. Food Funct. 9, 1510–1523. doi: 10.1039/C7FO01554B, PMID: 29423494

[ref108] Miljevic C. D. Nikolic-Kokic A. Blagojevic D. Milovanovic M. Munjiza A. Jukic M. M.. (2018). Association between neurological soft signs and antioxidant enzyme activity in schizophrenic patients. Psychiatry Res. 269, 746–752. doi: 10.1016/j.psychres.2018.09.009, PMID: 30273900

[ref109] Mishra A. Reeta K. H. Sarangi S. C. Maiti R. Sood M. (2022). Effect of add-on alpha lipoic acid on psychopathology in patients with treatment-resistant schizophrenia: a pilot randomized double-blind placebo-controlled trial. Psychopharmacology 239, 3525–3535. doi: 10.1007/s00213-022-06225-2 36069950 PMC9449282

[ref110] Mlyniec K. (2021). Interaction between zinc, GPR39, BDNF and neuropeptides in depression. Curr. Neuropharmacol. 19, 2012–2019. doi: 10.2174/1570159X19666210225153404, PMID: 33632103 PMC9185795

[ref111] Moreno J. Gaspar E. Lopez-Bello G. Juarez E. Alcazar-Leyva S. Gonzalez-Trujano E.. (2013). Increase in nitric oxide levels and mitochondrial membrane potential in platelets of untreated patients with major depression. Psychiatry Res. 209, 447–452. doi: 10.1016/j.psychres.2012.12.024, PMID: 23357685

[ref112] Moretti M. Budni J. Dos Santos D. B. Antunes A. Daufenbach J. F. Manosso L. M.. (2013). Protective effects of ascorbic acid on behavior and oxidative status of restraint-stressed mice. J. Mol. Neurosci. 49, 68–79. doi: 10.1007/s12031-012-9892-4 23054587

[ref113] Moretti M. Ribeiro C. M. Neis V. B. Bettio L. E. B. Rosa P. B. Rodrigues A. L. S. (2018). Evidence for the involvement of opioid system in the antidepressant-like effect of ascorbic acid. Naunyn Schmiedeberg's Arch. Pharmacol. 391, 169–176. doi: 10.1007/s00210-017-1446-4 29222646

[ref114] Moretti M. Rodrigues A. L. S. (2022). Functional role of ascorbic acid in the central nervous system: a focus on neurogenic and synaptogenic processes. Nutr. Neurosci. 25, 2431–2441. doi: 10.1080/1028415X.2021.1956848 34493165

[ref115] Moretti M. Werle I. da Rosa P. B. Neis V. B. Platt N. Souza S. V. S.. (2019). A single coadministration of subeffective doses of ascorbic acid and ketamine reverses the depressive-like behavior induced by chronic unpredictable stress in mice. Pharmacol. Biochem. Behav. 187:172800. doi: 10.1016/j.pbb.2019.172800, PMID: 31678791

[ref116] Munkholm K. Makinen I. J. O. Maigaard K. Coello K. Pagsberg A. K. Kessing L. V. (2024). Inflammatory and oxidative stress biomarkers in children and adolescents with bipolar disorder - a systematic review and meta-analysis. Neurosci. Biobehav. Rev. 163:105766. doi: 10.1016/j.neubiorev.2024.105766, PMID: 38885887

[ref117] Murphy M. P. Holmgren A. Larsson N. G. Halliwell B. Chang C. J. Kalyanaraman B.. (2011). Unraveling the biological roles of reactive oxygen species. Cell Metab. 13, 361–366. doi: 10.1016/j.cmet.2011.03.010, PMID: 21459321 PMC4445605

[ref118] Myken A. N. Ebdrup B. H. Sorensen M. E. Broberg B. V. Skjerbaek M. W. Glenthoj B. Y.. (2022). Lower vitamin C levels are associated with less improvement in negative symptoms in initially antipsychotic-naive patients with first-episode psychosis. Int. J. Neuropsychopharmacol. 25, 613–618. doi: 10.1093/ijnp/pyac029, PMID: 35532335 PMC9380709

[ref119] Neill E. Rossell S. L. Yolland C. Meyer D. Galletly C. Harris A.. (2022). N-acetylcysteine (NAC) in schizophrenia resistant to clozapine: a double-blind, randomized, placebo-controlled trial targeting negative symptoms. Schizophr. Bull. 48, 1263–1272. doi: 10.1093/schbul/sbac065, PMID: 35857752 PMC9673271

[ref120] Nery F. G. Tallman M. J. Cecil K. M. Blom T. J. Patino L. R. Adler C. M.. (2022). N-acetylcysteine for depression and glutamate changes in the left prefrontal cortex in adolescents and young adults at risk for bipolar disorder: a pilot study. Early Interv. Psychiatry 16, 195–199. doi: 10.1111/eip.13149 33797205

[ref121] Nie L. J. Liang J. Shan F. Wang B. S. Mu Y. Y. Zhou X. H.. (2021). L-carnitine and acetyl-L-carnitine: potential novel biomarkers for major depressive disorder. Front. Psych. 12:671151. doi: 10.3389/fpsyt.2021.671151, PMID: 34658942 PMC8514700

[ref122] Nisar R. Batool Z. Haider S. (2023). Electric foot-shock induces neurobehavioral aberrations due to imbalance in oxidative status, stress hormone, neurochemical profile, and irregular cortical-beta wave pattern in rats: a validated animal model of anxiety. Life Sci. 323:121707. doi: 10.1016/j.lfs.2023.121707, PMID: 37084951

[ref123] Oktay M. Asoglu M. Taskin S. Kirmit A. (2024). Biological markers in newly diagnosed generalized anxiety disorder patients: 8-OHdG, S100B and oxidative stress. Neuropsychiatr. Dis. Treat. 20, 19–24. doi: 10.2147/NDT.S444506, PMID: 38204917 PMC10778226

[ref124] Ozdal T. Sela D. A. Xiao J. Boyacioglu D. Chen F. Capanoglu E. (2016). The reciprocal interactions between polyphenols and gut microbiota and effects on bioaccessibility. Nutrients 8:78. doi: 10.3390/nu8020078 26861391 PMC4772042

[ref125] Papuc C. Goran G. V. Predescu C. N. Nicorescu V. Stefan G. (2017). Plant polyphenols as antioxidant and antibacterial agents for shelf-life extension of meat and meat products: classification, structures, sources, and action mechanisms. Compr. Rev. Food Sci. Food Saf. 16, 1243–1268. doi: 10.1111/1541-4337.12298 33371586

[ref126] Pasco J. A. Jacka F. N. Williams L. J. Evans-Cleverdon M. Brennan S. L. Kotowicz M. A.. (2012). Dietary selenium and major depression: a nested case-control study. Complement. Ther. Med. 20, 119–123. doi: 10.1016/j.ctim.2011.12.008, PMID: 22500660

[ref127] Paul B. D. Sbodio J. I. Snyder S. H. (2018). Cysteine metabolism in neuronal redox homeostasis. Trends Pharmacol. Sci. 39, 513–524. doi: 10.1016/j.tips.2018.02.007, PMID: 29530337 PMC5912966

[ref128] Phensy A. Driskill C. Lindquist K. Guo L. Jeevakumar V. Fowler B.. (2017). Antioxidant treatment in male mice prevents mitochondrial and synaptic changes in an NMDA receptor dysfunction model of schizophrenia. eNeuro 4:81. doi: 10.1523/ENEURO.0081-17.2017, PMID: 28819639 PMC5559903

[ref129] Raffa M. Atig F. Mhalla A. Kerkeni A. Mechri A. (2011). Decreased glutathione levels and impaired antioxidant enzyme activities in drug-naive first-episode schizophrenic patients. BMC Psychiatry 11:124. doi: 10.1186/1471-244X-11-124, PMID: 21810251 PMC3161936

[ref130] Raghu G. Berk M. Campochiaro P. A. Jaeschke H. Marenzi G. Richeldi L.. (2021). The multifaceted therapeutic role of N-acetylcysteine (NAC) in disorders characterized by oxidative stress. Curr. Neuropharmacol. 19, 1202–1224. doi: 10.2174/1570159X19666201230144109, PMID: 33380301 PMC8719286

[ref131] Ren X. Zou L. Zhang X. Branco V. Wang J. Carvalho C.. (2017). Redox signaling mediated by Thioredoxin and glutathione Systems in the Central Nervous System. Antioxid. Redox Signal. 27, 989–1010. doi: 10.1089/ars.2016.6925, PMID: 28443683 PMC5649126

[ref132] Rizzuto R. De Stefani D. Raffaello A. Mammucari C. (2012). Mitochondria as sensors and regulators of calcium signalling. Nat. Rev. Mol. Cell Biol. 13, 566–578. doi: 10.1038/nrm3412 22850819

[ref133] Rosenblat J. D. McIntyre R. S. (2016). Bipolar disorder and inflammation. Psychiatr. Clin. N. Am. 39, 125–137. doi: 10.1016/j.psc.2015.09.006 26876323

[ref134] Rossetti A. C. Paladini M. S. Riva M. A. Molteni R. (2020). Oxidation-reduction mechanisms in psychiatric disorders: a novel target for pharmacological intervention. Pharmacol. Ther. 210:107520. doi: 10.1016/j.pharmthera.2020.107520, PMID: 32165136

[ref135] Russo A. J. (2011). Decreased zinc and increased copper in individuals with anxiety. Nutr Metab Insights 4, 1–5. doi: 10.4137/NMI.S6349 23946656 PMC3738454

[ref136] Sanders L. L. O. de Souza Menezes C. E. Chaves Filho A. J. M. de Almeida Viana G. Fechine F. V. Rodrigues de Queiroz M. G. (2017). Alpha-lipoic acid as adjunctive treatment for schizophrenia: an open-label trial. J. Clin. Psychopharmacol. 37, 697–701. doi: 10.1097/JCP.0000000000000800 29053478

[ref137] Satala G. Duszynska B. Lenda T. Nowak G. Bojarski A. J. (2018). Allosteric inhibition of serotonin 5-HT(7) receptors by zinc ions. Mol. Neurobiol. 55, 2897–2910. doi: 10.1007/s12035-017-0536-0, PMID: 28455702 PMC5842505

[ref138] Sceneay J. Liu M. C. Chen A. Wong C. S. Bowtell D. D. Moller A. (2013). The antioxidant N-acetylcysteine prevents HIF-1 stabilization under hypoxia in vitro but does not affect tumorigenesis in multiple breast cancer models in vivo. PLoS One 8:e66388. doi: 10.1371/journal.pone.0066388, PMID: 23840457 PMC3688768

[ref139] Schiavone S. Sorce S. Dubois-Dauphin M. Jaquet V. Colaianna M. Zotti M.. (2009). Involvement of NOX2 in the development of behavioral and pathologic alterations in isolated rats. Biol. Psychiatry 66, 384–392. doi: 10.1016/j.biopsych.2009.04.033, PMID: 19559404

[ref140] Shadfar S. Parakh S. Jamali M. S. Atkin J. D. (2023). Redox dysregulation as a driver for DNA damage and its relationship to neurodegenerative diseases. Transl Neurodegener 12:18. doi: 10.1186/s40035-023-00350-4, PMID: 37055865 PMC10103468

[ref141] Shirai Y. Fujita Y. Hashimoto R. Ohi K. Yamamori H. Yasuda Y.. (2015). Dietary intake of Sulforaphane-rich broccoli sprout extracts during juvenile and adolescence can prevent phencyclidine-induced cognitive deficits at adulthood. PLoS One 10:e0127244. doi: 10.1371/journal.pone.0127244, PMID: 26107664 PMC4479552

[ref142] Singh N. Sharpley A. L. Emir U. E. Masaki C. Herzallah M. M. Gluck M. A.. (2016). Effect of the putative lithium mimetic ebselen on brain myo-inositol, sleep, and emotional processing in humans. Neuropsychopharmacology 41, 1768–1778. doi: 10.1038/npp.2015.343 26593266 PMC4770517

[ref143] Siwek M. Styczen K. Sowa-Kucma M. Dudek D. Reczynski W. Szewczyk B.. (2017). The serum concentration of copper in bipolar disorder. Psychiatr. Pol. 51, 469–481. doi: 10.12740/PP/OnlineFirst/65250, PMID: 28866717

[ref144] Slimen I. B. Najar T. Ghram A. Dabbebi H. Ben Mrad M. Abdrabbah M. (2014). Reactive oxygen species, heat stress and oxidative-induced mitochondrial damage. A review. Int. J. Hyperth. 30, 513–523. doi: 10.3109/02656736.2014.971446 25354680

[ref145] Sowa-Kucma M. Styczen K. Siwek M. Misztak P. Nowak R. J. Dudek D.. (2018). Are there differences in lipid peroxidation and immune biomarkers between major depression and bipolar disorder: effects of melancholia, atypical depression, severity of illness, episode number, suicidal ideation and prior suicide attempts. Prog. Neuro-Psychopharmacol. Biol. Psychiatry 81, 372–383. doi: 10.1016/j.pnpbp.2017.08.024, PMID: 28867391

[ref146] Supp A. D. Avila S. Jr. Mastella G. A. Damasio L. de Oliveira I. H. Godoi A. K.. (2021). Ascorbic acid supplementation attenuates schizophrenia-like symptoms in an animal model induced by ketamine. Int. J. Dev. Neurosci. 81, 26–36. doi: 10.1002/jdn.10058 32780510

[ref147] Szebeni A. Szebeni K. DiPeri T. Chandley M. J. Crawford J. D. Stockmeier C. A.. (2014). Shortened telomere length in white matter oligodendrocytes in major depression: potential role of oxidative stress. Int. J. Neuropsychopharmacol. 17, 1579–1589. doi: 10.1017/S1461145714000698, PMID: 24967945

[ref148] Tuon T. Meirelles S. S. de Moura A. B. Rosa T. Borba L. A. Botelho M. E. M.. (2021). Behavior and oxidative stress parameters in rats subjected to the animal's models induced by chronic mild stress and 6-hydroxydopamine. Behav. Brain Res. 406:113226. doi: 10.1016/j.bbr.2021.113226, PMID: 33684423

[ref149] Vasconcelos G. S. Ximenes N. C. de Sousa C. N. Oliveira Tde Q. Lima L. L. de Lucena D. F.. (2015). Alpha-lipoic acid alone and combined with clozapine reverses schizophrenia-like symptoms induced by ketamine in mice: participation of antioxidant, nitrergic and neurotrophic mechanisms. Schizophr. Res. 165, 163–170. doi: 10.1016/j.schres.2015.04.017, PMID: 25937462

[ref150] Veronese N. Stubbs B. Solmi M. Ajnakina O. Carvalho A. F. Maggi S. (2018). Acetyl-L-carnitine supplementation and the treatment of depressive symptoms: a systematic review and Meta-analysis. Psychosom. Med. 80, 154–159. doi: 10.1097/PSY.0000000000000537 29076953

[ref151] Wang W. Bai M. Jiang T. Li C. Li P. Zhou H.. (2019). Clozapine-induced reduction of l-carnitine reabsorption via inhibition/down-regulation of renal carnitine/organic cation transporter 2 contributes to liver lipid metabolic disorder in mice. Toxicol. Appl. Pharmacol. 363, 47–56. doi: 10.1016/j.taap.2018.11.007, PMID: 30465787

[ref152] Wang N. Liu X. Li X. T. Li X. X. Ma W. Xu Y. M.. (2021). 7,8-Dihydroxyflavone alleviates anxiety-like behavior induced by chronic alcohol exposure in mice involving tropomyosin-related kinase B in the amygdala. Mol. Neurobiol. 58, 92–105. doi: 10.1007/s12035-020-02111-0, PMID: 32895785

[ref153] Wang W. Lu Y. Xue Z. Li C. Wang C. Zhao X.. (2015). Rapid-acting antidepressant-like effects of acetyl-l-carnitine mediated by PI3K/AKT/BDNF/VGF signaling pathway in mice. Neuroscience 285, 281–291. doi: 10.1016/j.neuroscience.2014.11.025, PMID: 25463525

[ref154] Wang X. Qi Y. Zheng H. (2022). Dietary polyphenol, gut microbiota, and health benefits. Antioxidants (Basel) 11:1212. doi: 10.3390/antiox11061212 35740109 PMC9220293

[ref155] Wang J.-F. Shao L. Sun X. Young L. T. (2009). Increased oxidative stress in the anterior cingulate cortex of subjects with bipolar disorder and schizophrenia. Bipolar Disord. 11, 523–529. doi: 10.1111/j.1399-5618.2009.00717.x 19624391

[ref156] Wang J. Um P. Dickerman B. A. Liu J. (2018). Zinc, magnesium, selenium and depression: a review of the evidence, potential mechanisms and implications. Nutrients 10:584. doi: 10.3390/nu10050584, PMID: 29747386 PMC5986464

[ref157] Wen F. Tan Z. G. Xiang J. (2022). Cu-Zn SOD suppresses epilepsy in pilocarpine-treated rats and alters SCN2A/Nrf2/HO-1 expression. Epileptic Disord. 24, 647–656. doi: 10.1684/epd.2022.1434 35872622

[ref158] Wu S. Gao Q. Zhao P. Gao Y. Xi Y. Wang X.. (2016). Sulforaphane produces antidepressant- and anxiolytic-like effects in adult mice. Behav. Brain Res. 301, 55–62. doi: 10.1016/j.bbr.2015.12.030, PMID: 26721468

[ref159] Wu P. F. Guan X. L. Wang F. Chen J. G. (2022). N-acetylcysteine facilitates extinction of cued fear memory in rats via reestablishing basolateral amygdala glutathione homeostasis. Acta Pharmacol. Sin. 43, 260–272. doi: 10.1038/s41401-021-00661-0, PMID: 33927360 PMC8791957

[ref160] Wu P. F. Han Q. Q. Chen F. F. Shen T. T. Li Y. H. Cao Y.. (2021). Erasing m(6)A-dependent transcription signature of stress-sensitive genes triggers antidepressant actions. Neurobiol Stress 15:100390. doi: 10.1016/j.ynstr.2021.100390, PMID: 34527794 PMC8430387

[ref161] Xiong R. G. Li J. Cheng J. Zhou D. D. Wu S. X. Huang S. Y.. (2023). The role of gut microbiota in anxiety, depression, and other mental disorders as well as the protective effects of dietary components. Nutrients 15:258. doi: 10.3390/nu15143258, PMID: 37513676 PMC10384867

[ref162] Yang Y. S. Maddock R. J. Zhang H. Lee J. Hellemann G. Marder S. R.. (2022). N-acetylcysteine effects on glutathione and glutamate in schizophrenia: a preliminary MRS study. Psychiatry Res. Neuroimaging 325:111515. doi: 10.1016/j.pscychresns.2022.111515, PMID: 35839558

[ref164] Yatham L. N. Kennedy S. H. Parikh S. V. Schaffer A. Bond D. J. Frey B. N.. (2018). Canadian network for mood and anxiety treatments (CANMAT) and International Society for Bipolar Disorders (ISBD) 2018 guidelines for the management of patients with bipolar disorder. Bipolar Disord. 20, 97–170. doi: 10.1111/bdi.12609, PMID: 29536616 PMC5947163

[ref165] Yin R. Mao S. Q. Zhao B. Chong Z. Yang Y. Zhao C.. (2013). Ascorbic acid enhances Tet-mediated 5-methylcytosine oxidation and promotes DNA demethylation in mammals. J. Am. Chem. Soc. 135, 10396–10403. doi: 10.1021/ja4028346 23768208

[ref166] Yumru M. Savas H. A. Kalenderoglu A. Bulut M. Celik H. Erel O. (2009). Oxidative imbalance in bipolar disorder subtypes: a comparative study. Prog. Neuro-Psychopharmacol. Biol. Psychiatry 33, 1070–1074. doi: 10.1016/j.pnpbp.2009.06.005, PMID: 19527764

[ref167] Zeng T. Li J. Xie L. Dong Z. Chen Q. Huang S.. (2023). Nrf2 regulates iron-dependent hippocampal synapses and functional connectivity damage in depression. J. Neuroinflammation 20:212. doi: 10.1186/s12974-023-02875-x, PMID: 37735410 PMC10512501

[ref168] Zeng J. Zhang W. Lu X. Zhou H. Huang J. Xu Z.. (2024). The association of SOD and HsCRP with the efficacy of sulforaphane in schizophrenia patients with residual negative symptoms. Eur. Arch. Psychiatry Clin. Neurosci. 274, 1083–1092. doi: 10.1007/s00406-023-01679-7, PMID: 37728803 PMC11226471

[ref169] Zhang J. C. Yao W. Dong C. Yang C. Ren Q. Ma M.. (2015). Comparison of ketamine, 7,8-dihydroxyflavone, and ANA-12 antidepressant effects in the social defeat stress model of depression. Psychopharmacology 232, 4325–4335. doi: 10.1007/s00213-015-4062-3, PMID: 26337614

